# mHealth Apps in German Outpatient Mental Health Care: Protocol for a Mixed Methods Approach

**DOI:** 10.2196/56205

**Published:** 2024-12-10

**Authors:** Klemens Höfer, Felix Plescher, Sarah Schlierenkamp, Stefanie Solar, Silke Neusser, Udo Schneider, Dieter Best, Jürgen Wasem, Carina Abels, Anna Bußmann

**Affiliations:** 1 Institute for Healthcare Management and Research University of Duisburg-Essen Essen Germany; 2 Essener Forschungsinstitut für Medizinmanagement GmbH Essen Germany; 3 Techniker Krankenkasse Hamburg Germany; 4 Deutsche PsychotherapeutenVereinigung e.V. Berlin Germany

**Keywords:** mental health care, mHealth, digital health applications, Digitale Gesundheitsanwendungen, mental disorders, health care research, applications, app, mental health, outpatient, digital health, adults, Germany, mobile health apps, treatment

## Abstract

**Background:**

Mental disorders are complex diseases that affect 28% (about 17.8 million people) of the adult population in Germany annually. Since 2020, certain mobile health (mHealth) apps, so-called digital health applications (DiGA), are reimbursable in the German statutory health insurance system. A total of 27 of the 56 currently available DiGA are approved for the treatment of mental and behavioral diseases. An indicator of existing problems hindering the use of DiGA is the rather hesitant prescribing behavior.

**Objective:**

This project aims to develop health policy recommendations for the optimal integration of DiGA into outpatient psychotherapeutic care. The project is funded by the Innovation Fund of the Joint Federal Committee (grant 01VSF22029). The current status quo of the use of DiGA will be analyzed. Furthermore, concepts for the integration of mHealth apps, as well as their transfer into the care process will be investigated. In addition, barriers will be identified, and existing expectations of different perspectives captured.

**Methods:**

The project will be based on a mixed methods approach. A scoping review and a qualitative analysis of focus groups and expert interviews will be carried out. Additionally, an analysis of claims data of the statutory health insurance will be conducted. This will be followed by a written survey of insured persons and health care providers. Finally, health policy recommendations will be derived in cooperation with stakeholders.

**Results:**

The scoping reviews and qualitative analyses have been completed, and the quantitative surveys are currently being carried out. The target number of responses in the survey of insured persons has already been achieved. Furthermore, the analysis claims data of the statutory health insurance is currently being conducted.

**Conclusions:**

There is a need for research on how DiGA can be optimally integrated into the care process of patients with mental disorders as evidence regarding the topic is limited and prescribing behavior low. Although the potential of DiGA in mental health care has not yet fully unfolded, Germany serves as a model for other countries regarding reimbursable mHealth apps. This project aims to explore the potentials of DiGA and to describe the organizational, institutional, and procedural steps necessary for them to best support mental health care.

**International Registered Report Identifier (IRRID):**

DERR1-10.2196/56205

## Introduction

### Background

Every year, approximately 28% of the adult population in Germany (approximately 17.8 million people) is affected by a mental disorder. Their prevalence has increased in the recent years [1]. Among these complex conditions, the most common include anxiety disorders, mood disorders, and disorders relating to substance misuse [2]. For patients and their relatives, mental illnesses cause significant personal and health-related burdens [3]. Further, mental disorders can lead to major challenges for society, such as prolonged sick leave. For example, Germany’s largest statutory health insurance (SHI) company—Techniker Krankenkasse (TK)—found that 20% of all absences at work could be attributed to mental disorders in 2020, which is the highest level since records began [4].

In Germany, a multifaceted support and health care system is in place for people having from mental disorders. The treatment costs are predominantly borne by SHI. Depending on the illness and its burdens, easily accessible psychosocial interventions, psychotherapeutic treatments and other types of therapy are available to patients [5]. However, when it comes to the psychotherapeutic treatment itself, issues relating to access and interface problems can arise. Insured persons wanting to access outpatient psychotherapy often have to contact various psychotherapists before finding long-term therapy provisions. This requires skills such as health literacy and self-management. These skills can help individuals to make the necessary efforts. Since not all potential patients have these competencies, some groups are disadvantaged, and therefore, at risk of disease progression up to a chronic level [6].

Mobile health (mHealth) apps represent one possibility for reducing problems associated with access and improving the health care provision currently available [7]. In this paper, smartphone apps, as well as browser applications, are meant by this term. They provide an easily accessible option for improving access to treatment, especially in the case of disorders that are often accompanied by stigmatization and shame, such as mental disorders. Moreover, mHealth apps can be used to support outpatient treatment and be integrated into relevant processes [8].

Since the Digital Act (Digitale-Versorgungs-Gesetz) in 2019, health care providers in Germany have been able to prescribe reimbursable digital health applications (Digitale Gesundheitsanwendungen or DiGA in German) also known as “apps on prescription.” In September 2020, the first DiGA was approved for inclusion in the official DiGA directory of the Federal Institute for Drugs and Medical Devices [9]. DiGA are a digital-technology-based medical device (Medical Device Regulation—Class I, IIa or IIb) [10]. In principle, a DiGA may be either a native app or a desktop or browser application. DiGA can also include other hardware such as virtual reality glasses. These apps support the detection, monitoring, treatment, and alleviation of medical conditions but are not permitted for use as primary prevention [10]. For mHealth apps for mental disorders to be reimbursed by the SHI in Germany, they must undergo an assessment process and prove a positive impact on health care. This positive impact may be either a medical benefit or a patient-relevant structural or procedural improvement [10]. The so-called “Fast Track Process” is an initial admission procedure for inclusion within the DiGA directory, which allows them to be reimbursed by the SHI [10]. This process requires potential DiGA to prove their positive health care impact through comparative studies. As a minimum standard, retrospective studies are required. However, in most cases, randomized controlled trials are carried out in order to fulfill this evidence requirement [10].

mHealth apps in general, and DiGA specifically, enable new approaches to and methods of treatment, in which the patients become independent actors with regard to the management of their illnesses. These apps have the potential to enhance the insured person’s ability to self-manage and reflect on their illness, for example, by symptom recording and awareness exercises [11]. In addition to allowing patients to proactively influence their treatment, data, and information collected by mHealth apps and DiGA can also be used to optimize existing treatment processes and reduce liaison issues between different health care sectors potentially enhancing the quality of care provided [12].

However, the use of DiGA is still relatively infrequent in Germany. One projection has revealed that only 374,377 DiGA codes had been generated by September 2023 [13]. Further, the potential of DiGA is restricted by a high user dropout rate. Studies observing people with chronic illnesses have estimated dropout rates of up to 43% [14]. Barriers to fully using the potential of mHealth apps include poor user-friendliness, patient fears concerning insufficient contact with a health care provider, and apps’ limited ability to be adapted to patients’ individual needs [14].

### Project Objective

The objective of the project “Realizing the Potential of Digital Health Applications (DiGA) in Outpatient Mental Health Care (DiGAPsy)” is to develop public health policy recommendations—in terms of a blueprint—for optimizing the incorporation of DiGA within mental health care. The project is funded by the Innovation Fund of the German Federal Joint Committee (grant 01VSF22029). One focus of the project will be to combine the use of DiGA with traditional components of outpatient (mental) health care. This will include looking at the potential of DiGA as a stand-alone treatment—for example, for mild mental disorders—as well as the potential of bridging waiting times and the possibility of integrating DiGA within the psychotherapeutic and psychiatric treatment process itself. The project will also examine how and in which situations DiGA can be used. Different options for the integration of mHealth apps such as a substitute or a supplement to traditional forms of care will be analyzed. Ensuring an optimized incorporation of DiGA within health care provision is essential. To achieve this, it is necessary to mitigate existing provision deficits and to prevent problems relating to unsuitable use of DiGA, as well as liaison issues. This research aims to promote both the purposeful deployment of DiGA and a patient-centered approach to the incorporation of DiGA within health care provision to improve the quality of care.

### Research Questions

This project will address the following research questions:

- What approaches to the incorporation of mHealth apps within mental health care exist nationally and internationally? What are the potential areas for application in terms of outpatient mental health care?
- What are the current barriers preventing DiGA from being implemented and used by health care providers (the term “health care providers” refers to both physicians and psychotherapists) and patients both in Germany and internationally?
- How are DiGA currently used for the treatment of patients with mental disorders? (Status quo)
  Can key areas of application be identified? Who is prescribing DiGA, what indications are DiGA prescribed for, under which circumstances are they prescribed, and for what type of patients?
  What is the perspective of patients and health care providers on the integration of DiGA within the treatment process?
    Within which diagnostic and therapeutic treatments are DiGA used and how often (number of follow-up prescriptions)? (Prescription patterns)
    For what purpose, at which stage of the illness, and for how long are DiGA used by patients? (Use patterns) Can specific patient journeys be identified?
    Under what circumstances do patients use DiGA without a prescription from their health care provider?
- What are health care providers’ and patients’ preferences when it comes to the design, areas of application, and organizational structures of DiGA and what expectations do both groups have in terms of the integration of these apps within mental health care? What barriers prevent a more extensive incorporation of DiGA within mental health care and at which points of the care provision process do these barriers arise?
- What recommendations can be derived based on the results both for integrating DiGA within mental health care and for ensuring good coordination between the DiGA and the various health care providers?
  Which adjustments are necessary in different care settings to sustainably and economically realize the innovative potentials of DiGA for patients with mental disorders?
  How can these viable recommendations be institutionally and procedurally implemented? (Implementation)

Recommendations derived from answering these questions will outline the organizational, institutional, and procedural steps necessary to ensure that DiGA provide optimized support for the treatment of patients with mental disorders. These recommendations will determine how these apps can be used in a manner that maximizes their potential effectiveness.

## Methods

### Overview

**Figure 1:**
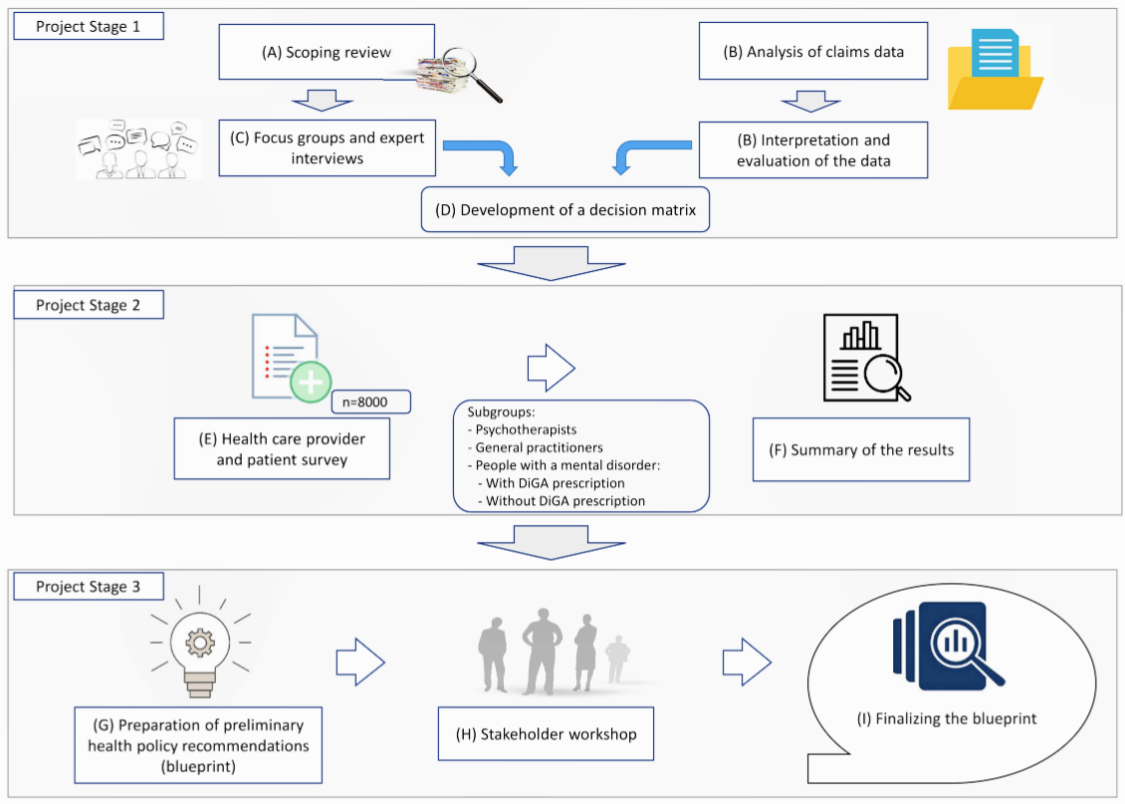
Project overview—DiGAPsy.

A mixed methods approach will be used based on three different complementary and consecutive project stages. The mixture of research methods enables to integrate all relevant perspectives on the research topic into the project. Relevant approaches to the integration of mHealth apps within mental health care will be identified and the transferability to the German health care system examined. In addition, current treatment practice, as well as the barriers preventing more extensive incorporation of DiGA, will also be assessed. Based on this information, recommendations for optimizing the incorporation of DiGA within mental health care will subsequently be derived with the ultimate objective of improving the quality of care. Figure 1 illustrates the study design.

### Project Stage 1

#### Overview

Project stage 1 will examine research questions 1 to 3 and is divided into four modules. The results from project stage 1 will function as a base for the analytical steps to be carried out in project stages 2 and 3.

#### Module A: Scoping Review

The first module aims to identify national and international evidence regarding (1) approaches to the integration of DiGA and DiGA-equivalent mHealth apps within mental health care and (2) the barriers that hinder the optimized incorporation of these apps within outpatient care provision. The objective is to identify best practice models and the relevant parameters for the implementation of DiGA.

Therefore, a scoping review will be carried out [15] and for compilation and reporting purposes, the PRISMA-ScR (Preferred Reporting Items for Systematic reviews and Meta-Analyses extension for Scoping Reviews) checklist will be used [16]. Title and abstract screening, as well as the full-text screening, will be carried out by two researchers (FP and SSolar) independently of each other.

The identified approaches to the incorporation of DiGA within mental health care will be used as a base for the subsequent development of a decision matrix. The results of the scoping review will be published in a subject-related journal.

#### Module B: Analyzing Claims Data of the SHI

This module aims to analyze the prescription and use patterns, as well as the current incorporation of DiGA treatment pathways. Data for the analysis are based on SHI claims data of the TK. The TK is the largest German SHI in terms of number of insured persons, providing insurance to 11.2 million people [17].

The analysis will include data of adult insured persons of 18 years or older who received an ICD-10-GM (German Modification of the International Statistical Classification of Diseases and Related Health Problems) F-diagnosis (mental and behavioral disorders) and a DiGA in the category of mental disorders during the index period. A matching analysis is carried out to compare patient journeys. For this purpose, a control group consisting of people older than 18 years who have received an *ICD-10* (*International Statistical Classification of Diseases, Tenth Revision*) F-diagnosis and no DiGA is included.

**Figure 2:**
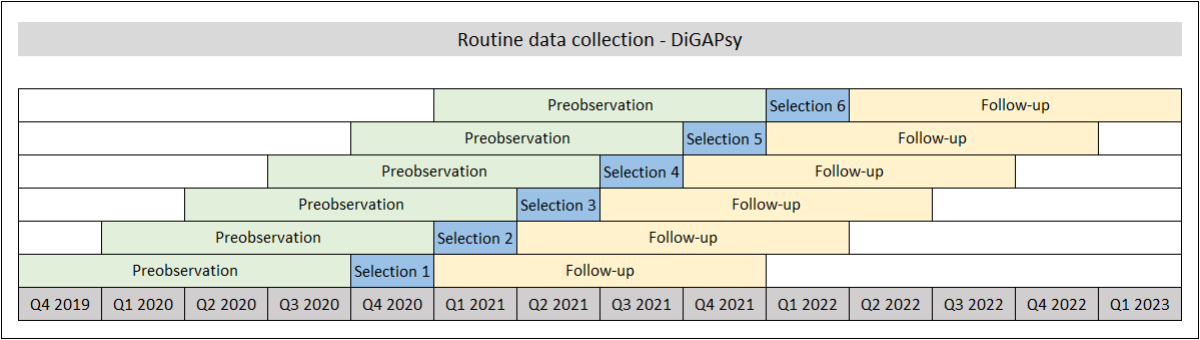
Routine data collection - DiGAPsy.

The index period will be from Q4 2020 to Q1 2022. The observation period for the included persons will cover a total of 2 years: 12 months prior to the DiGA and 12 months subsequent to this. Figure 2 illustrates the planned procedure.

A descriptive and multivariate analysis of current key areas of application, prescription, and use patterns, as well as patient journeys, will be carried out, taking into consideration aspects such as patient characteristics and use of other health care options. Furthermore, the patient journeys and patient characteristics will be compared to those of non-DiGA users identified by a matching approach.

Univariate and bivariate analyses will be carried out. Depending on the variable, frequencies, mean or median values will be compared, alongside statistical significance testing. Subgroup analyses will also be performed, for example, based on different diagnosis indications.

#### Module C: Focus Groups and Expert Interviews

The objective of this module is to answer research questions 1 (approaches, best practice, potential areas of application) and 2 (barriers), factoring in results from the scoping review (module A).

To consider the perspective of psychotherapists and physicians, as well as of patients (DiGA-using and non–DiGA-using), 8 focus groups of approximately 6 persons will be carried out. Additionally, 12 semistructured interviews will be conducted to incorporate other relevant perspectives, such as those of medical associations, SHI companies, and DiGA developers. To motivate individuals to participate, financial incentives will be provided. A declaration of informed consent has to be signed by participants to ensure voluntary participation and confidentiality.

Following Krueger and Casey [18], the focus groups and interviews will be guided by a team of moderators. These will be based on semistructured interview guides, which will allow the integration of findings from the literature review (module A) and provide flexibility to explore any additional aspects that have not yet been specifically identified. The guides will be adapted to the specific target groups, whilst also attempting to ensure results demonstrate a certain level of comparability.

The focus groups and interviews will primarily take place via a videoconferencing system. There will be a video recording, which will be transcribed afterward. A qualitative content analysis will then be carried out following Mayring and Kuckartz using the software MAXQDA (VERBI GmbH) [19,20].

#### Module D: Development of a Decision Matrix

The objective of this module is to systematically summarize the project results from module A (scoping review) and module C (focus groups and interviews).

Module D will result in the establishment of a 2D decision matrix for preparing the policy recommendations. Furthermore, the matrix will enable best practice models to be examined in the context of existing barriers both of which will have been identified in modules A and C. Subsequently, the results from the claims data analysis will be incorporated into the decision matrix (module B).

### Project Stage 2

#### Overview

Project stage 2 will implement modules E and F to address research questions 2 to 4 (barriers, status quo, and expectations). The results from project stage 1, particularly the decision matrix from module D, will be used for drafting the survey.

#### Module E: Patient and Health Care Provider Survey

##### Overview

This module will involve collecting primary data using a survey questionnaire. The target groups for this survey will be patients insured by TK who have mental disorders (group A) and health care providers (group B). Both surveys will be carried out anonymously. Respondents will be informed about the study and the voluntariness of participation. For the written surveys, samples of at least 201 respondents will be necessary to determine statistically significant mean differences with a power of 90% and α value of .05 for a moderate effect size for the four groups (groups A1, A2, B1, and B2). Accordingly, 2000 people per group will be contacted.

##### Patient Survey (Group A)

Two patient groups will be analyzed. DiGA users will be surveyed to collect information relating to the use and incorporation of DiGA within health care provision from the perspective of the patient. Additionally, nonusers will be included to analyze potential barriers. Both groups will be recruited by the TK according to the inclusion and exclusion criteria:

- Group A1: adults who will have received reimbursement for at least one DiGA for an *ICD-10* F-diagnosis in Q1 2023.
- Group A2: adults with a relevant *ICD-10* F-diagnosis who will have not received a DiGA reimbursement in 2022 or 2023.

Further inclusion and exclusion criteria will be defined by the consortium based on the results already obtained by the project.

The insured persons will each receive a questionnaire adapted to the specifics of their respective group (group A1 or A2). Participation will be possible by filling out the questionnaire manually or digitally.

##### Health Care Provider Survey (Group B)

To achieve a comprehensive overview both of health care provision and potential barriers, 2 groups of certified health care providers authorized by the SHI will be recruited:

- Group B1: psychotherapists and psychiatrists
- Group B2: general practitioners

For this, a random sample of groups B1 and B2 that meet the requirements will be selected by an agency. The exact inclusion and exclusion criteria will be defined by the consortium based on the results already obtained by the project. The survey questionnaires will be adapted according to the specifics of the two different groups.

##### Questionnaire Development

The questionnaires will be developed based on the results of project stage 1, module D. For this, current guidance and recommendations will be taken into account [5,21]. Sociodemographic and socioeconomic factors will be surveyed to identify any differences between patient groups or health care providers.

##### Interpretation

The survey will be designed to provide results on how DiGA are incorporated within mental health care, the impact of DiGA use on the quality of health care provision, difficulties that patients and health care providers encounter regarding the incorporation of DiGA within patients’ specific treatment, as well as respondents’ expectations. In addition, the survey will record how DiGA impact mental health care–related liaison issues between different health care sectors.

#### Module F: Summary of the Results

Upon completion of the second project stage, results will be summarized. This will involve integrating the results from the survey and the finalized claims data analysis into the decision matrix from project stage 1, module D. The barriers identified during the scoping review, focus groups, and interviews will be reviewed and verified for the German perspective using the results of the surveys. The combined results from the extended decision matrix will then be discussed with and approved by the project consortium. The results will be included in a scientific paper and be used as a basis for project stage 3.

### Project Stage 3

#### Overview

Project stage 3 consists of 3 consecutive modules and pursues the objective of answering research question 5 (health policy recommendations). It aims to derive a comprehensive strategy based on the matrix of module F to improve the integration of DiGA in outpatient mental health care.

#### Module G: Preparation of Preliminary Health Policy Recommendations (Blueprint)

The objective of module G is to develop actionable health policy recommendations for optimizing the incorporation of DiGA within mental health care. This will involve establishing recommendations relating to legislation or sublegislative regulations.

#### Module H: Stakeholder Workshop

The consistency and applicability of the conclusions reached in module G will be examined during a critical discussion, bringing together the perspectives of a variety of different stakeholders, such as Medical Associations, the Association of SHI Physicians, the Federal Institute for Drugs and Medical Devices, and SHI companies. In terms of an expert workshop, a maximum of 20 relevant stakeholders will discuss the conclusions of the blueprint (module G) to find consent or argue about substantiate dissent.

#### Module I: Finalizing the Blueprint

The blueprint will be adapted and finalized in accordance with the results of the workshop, producing a coherent and practically applicable overall strategy to better incorporate DiGA into outpatient mental health care, which can be implemented within the SHI system.

### Ethical Considerations

The study received ethical approval from the Ethics Committee of the Medical Faculty of the University of Duisburg-Essen on April 2, 2024 (reference: 23-11209-BO). For the purpose of the claims data analysis (module B), pseudonymized data will be used, assuring that there is no possibility of participant identification. Data protection and safety are ensured by a data protection concept, which is approved by the Federal Social Security Office. The approval allows to analyze the SHI claims data without additional informed consent. The data collected in the focus groups and interviews (module C) is considered pseudonymized because, after the transcription, all personal data will be removed. Before participation, participants will need to sign informed consent which allows the transcripts to be stored and analyzed. The list of participants will be deleted after conducting the focus groups. The audio recordings will be deleted after transcription. Participants in the survey (module E) will be informed that their participation is voluntary and anonymous and that they agree to participate by submitting the survey. To take part in the workshop (module H), participants will need to sign an informed consent. Participants will receive financial incentives for participating in the focus groups, interviews, and workshops.

## Results

This project will examine the incorporation of DiGA within mental health care. It aims to improve the current integration and use of DiGA. It will also identify the requirements for a successful implementation of DiGA in the health care system. This will be done by analyzing empirical data from a variety of sources and including the perspectives of health care providers and (potential) patients. This way, targeted, realistic, and empirically based health policy recommendations for legislators and self-governing bodies will be developed. These recommendations shall support the development of an appropriate and economical use of DiGA in mental health care. Concepts for the integration of mHealth apps for mental disorders will be derived and potential areas of application in the care process will be defined. The project will examine the status quo of how DiGA are used in outpatient psychotherapeutic care (key areas of application, prescription patterns, use patterns, and patient journeys). Further, conclusions will be drawn concerning barriers that prevent a more extensive implementation of DiGA. The circumstances under which health care providers recommend the use of DiGA and other facilitating factors will be analyzed. Expectations regarding format, content, and usability will also be part of the study. Further, the conditions under which patients are most likely to accept the use of these apps as part of their psychotherapeutic treatment will be analyzed. These recommendations for the deployment of DiGA within mental health care will be presented in a blueprint and thus be available to a professional readership (eg, health care providers and other relevant stakeholder groups). The knowledge obtained through this research project has the potential to contribute to the improvement and adaptation of health care based on patient’s needs. As of November 2024, the scoping review and qualitative analyses have been completed, and the quantitative surveys are currently being carried out. The target number of responses in the survey of insured persons has already been achieved. Furthermore, the analysis of claims data for the SHI is currently being conducted.

## Discussion

### Principal Findings

The aim of the project “DiGAPsy” is to optimize the integration of DiGA in mental health care. At the end of the project, health policy recommendations for an optimal integration of DiGA in outpatient mental health care will be developed. A mixed methods approach will be used, combining both qualitative and quantitative methods.

The use of DiGA, reimbursed by the SHI, has been possible since the end of 2020. Therefore, there is a limited amount of evidence regarding the topic. Germany was the first country to introduce reimbursable mHealth apps in the health care system and thus serves as a role model for other countries considering the implementation of DiGA-similar concepts. The innovative character of DiGA limits the extent to which international studies can be found. Existing international studies such as surveys on preferences for mHealth apps have only analyzed individual aspects of the issue, such as existing problems and barriers related to the use of DiGA in general [22]. Another aspect was analyzed in a study by Smail-Crevier et al [23], where they showed that gender differences in opinions exist. Their study showed that women were more likely than men to use mHealth apps and seek health information digitally. Men preferred programs with a high degree of gamification.

This study aims to optimize the integration of DiGA into outpatient mental health care and fill research gaps. mHealth apps for mental disorders—including DiGA—are burdened by limiting factors and a range of barriers hindering their optimal integration, which reduces their usefulness in health care [24,25]. As a result, mHealth apps and DiGA tend to fall short of expectations and fail to fully unfold their potential. This is particularly problematic in the German scope, as the financial situation of the German health care system is worsening and there is a shortage of skilled workers. Innovative approaches are needed to improve the efficiency of the health care system. The project presented here provides concrete, actionable recommendations for a holistic and patient-centered use of DiGA, which help to overcome existing obstacles. This will help to improve the quality and efficiency of the health care system.

### Limitations

Limitations include that this study focuses on mHealth apps that are reimbursable by the German statutory health care system (DiGA). The framework of financing and eligibility for reimbursement of certified mHealth apps may be different in other countries. Thus, the results of this study may not be completely applicable to them. As there are different legal frameworks, there may be also other requirements for reimbursable certified mHealth apps. For example, other countries might have a different perspective on data protection requirements. This could lead to the addition of blended care features, which are currently not considered in the concept of DiGA. Another limitation is that the level of DiGA integration within mental health care is still relatively low. Low prescription numbers demonstrate hesitancy when it comes to prescribing DiGA across all indications. Similarly, despite half of approved DiGA being for mental disorders, the highest prescription rates of DiGA can be found for obesity, back pain, and tinnitus [13].

### Conclusions

The results of this study do not only aim to improve the implementation of DiGA in outpatient mental health care in Germany but may be also of international interest. Even if different legal frameworks may limit the direct application of the study results in other countries, they provide a starting point for the design of blueprints to facilitate the integration of similar apps. Thus, the knowledge and research results can help implement similar systems within their health care structures. This helps to avoid mistakes and accelerate implementation. France and Austria, for instance, consider rolling out mHealth apps using a similar strategy [26].

## Abbreviations

DiGA: Digitale Gesundheitsanwendungen
*ICD-10*: *International Statistical Classification of Diseases, Tenth Revision*
ICD-10-GM: German Modification of the International Statistical Classification of Diseases and Related Health Problems
mHealth: mobile health
PRISMA-ScR: Preferred Reporting Items for Systematic reviews and Meta-Analyses extension for Scoping Reviews
SHI: statutory health insurance
TK: Techniker Krankenkasse
